# The Anticipated Severity of a “1918-Like” Influenza Pandemic in Contemporary Populations: The Contribution of Antibacterial Interventions

**DOI:** 10.1371/journal.pone.0029219

**Published:** 2012-01-23

**Authors:** Yu-Wen Chien, Bruce R. Levin, Keith P. Klugman

**Affiliations:** 1 Department of Epidemiology, Rollins School of Public Health, Emory University, Atlanta, Georgia, United States of America; 2 Department of Biology, Emory University, Atlanta, Georgia, United States of America; 3 Hubert Department of Global Health, Rollins School of Public Health, Emory University, Atlanta, Georgia, United States of America; Albany Medical College, United States of America

## Abstract

Recent studies have shown that most of deaths in the 1918 influenza pandemic were caused by secondary bacterial infections, primarily pneumococcal pneumonia. Given the availability of antibiotics and pneumococcal vaccination, how will contemporary populations fare when they are next confronted with pandemic influenza due to a virus with the transmissibility and virulence of that of 1918? To address this question we use a mathematical model and computer simulations. Our model considers the epidemiology of both the influenza virus and pneumonia-causing bacteria and allows for co-infection by these two agents as well as antibiotic treatment, prophylaxis and pneumococcal vaccination. For our simulations we use influenza transmission and virulence parameters estimated from 1918 pandemic data. We explore the anticipated rates of secondary pneumococcal pneumonia and death in populations with different prevalence of pneumococcal carriage and contributions of antibiotic prophylaxis, treatment, and vaccination to these rates. Our analysis predicts that in countries with lower prevalence of pneumococcal carriage and access to antibiotics and pneumococcal conjugate vaccines, there would substantially fewer deaths due to pneumonia in contemporary populations confronted with a 1918-like virus than that observed in the 1918. Our results also predict that if the pneumococcal carriage prevalence is less than 40%, the positive effects of antibiotic prophylaxis and treatment would be manifest primarily at of level of individuals. These antibiotic interventions would have little effect on the incidence of pneumonia in the population at large. We conclude with the recommendation that pandemic preparedness plans should consider co-infection with and the prevalence of carriage of pneumococci and other bacteria responsible for pneumonia. While antibiotics and vaccines will certainly reduce the rate of individual mortality, the factor contributing most to the relatively lower anticipated lethality of a pandemic with a 1918-like influenza virus in contemporary population is the lower prevalence of pneumococcal carriage.

## Introduction

Dominating our fears, driving our surveillance efforts and preparations for preventing, limiting the spread and treating influenza is the “Mother of all pandemics,” the1918 flu [1]. Never in recorded history has the world confronted a single infectious disease pandemic that lead to as many deaths; estimates ranging from 20–100 million for the world at large, and on the order of 675,000 in the United States alone [2], [3], [4]. An estimated 28% of Americans were symptomatically infected by this virus [2] and, unlike most influenza pandemics, the rate of mortality was particularly high in people in their prime of life, those aged 18–40 years [1].

Can it happen again? Evidence from virus reconstruction and animal model experiments suggests that the H1N1 influenza virus responsible for the 1918 flu was more virulent than contemporary viruses of this type of hemagglutinin and neuraminidase [3], [4], [5], [6]. While we may not be able to say when, there is every reason to expect that the mutation and recombination events responsible for the evolution of influenza viruses with the combination of the virulence, and human to human transmissibility of the 1918 flu can and doubtless will be repeated.

Given what we know now about the 1918 influenza pandemic and the medical and public health technology currently available, in contemporary human populations what would be the incidence of symptomatic infections and the mortality rate of a pandemic with an influenza virus of the virulence and transmissibility of that of 1918? What would be the optimum procedure to deal with this potential pandemic?

To address these questions, we use a mathematical model and computer simulations. Central to our model and analysis is the evidence that most of the pneumonias and deaths of the 1918 influenza pandemic can be attributed to a kind of conspiracy between the influenza virus and bacteria, primarily secondary infections with *Streptococcus pneumoniae* [7], [8], [9]. As evidence now indicates [10], [11], in our co-infection model individuals infected both with the influenza virus and the bacteria have higher rates of mortality than those infected with the virus or bacteria alone. We calibrate our model by exploring the conditions required for it to account for dynamics and mortality rates observed in 1918, using virus transmission, pneumococcal carriage and virulence parameters estimated from the most realiable1918 data we can find. We then consider the incidence and mortality rates of secondary pneumococcal pneumonia that would be anticipated for a pandemic with a virus of the 1918 ilk with the pneumococcal carriage prevalence of contemporary populations in developed and developing countries, and with antibiotics for prophylaxis and treatment of secondary bacterial pneumonia. We further consider the impact of pneumococcal conjugate vaccination of infants, which has been shown to reduce hospitalization due to influenza [12], [13]. We discuss the implications of these computer simulation results to planning for the next influenza pandemic.

## Methods

### Model development

Our complete “compartment” model [14] including co-infection with the influenza virus and bacteria; and antibiotic prophylaxis and treatment of the bacterial infection is obviously complex. To facilitate its presentation, we separately consider its different components and how they are modeled.

#### i) Single infection with the pandemic influenza virus

**Figure 1:**
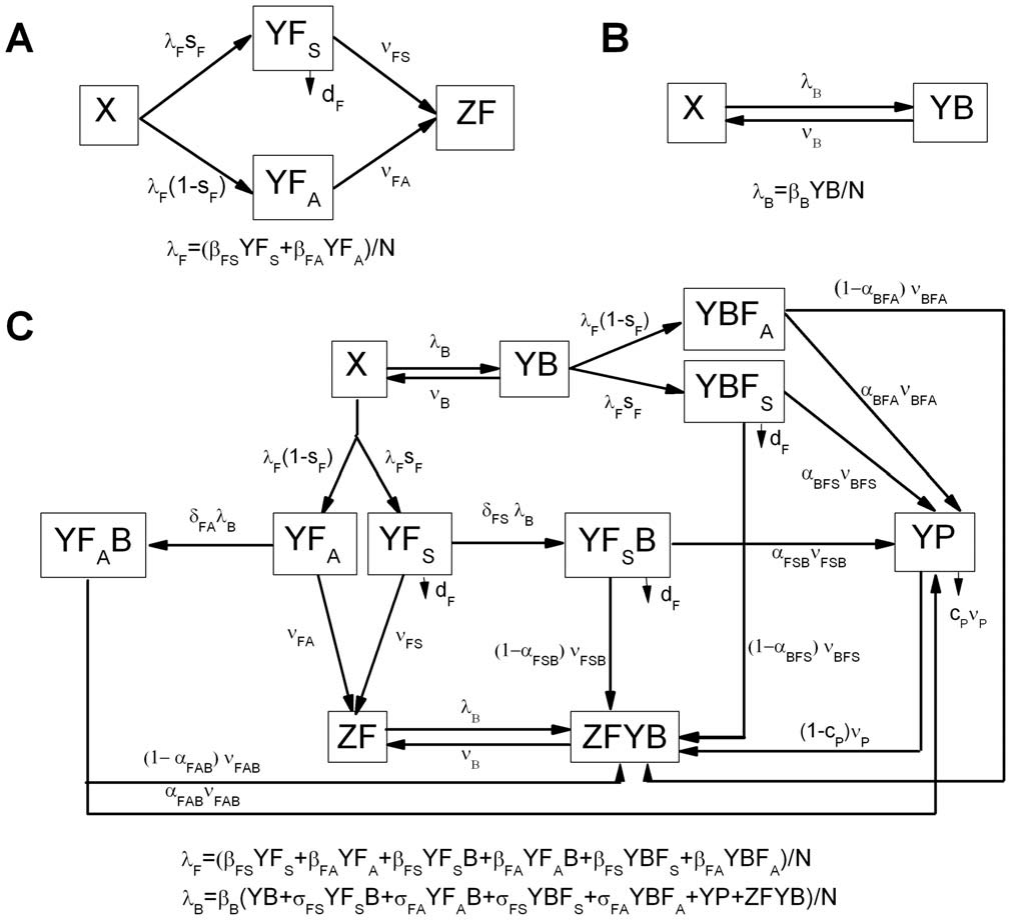
Model structure. (A) Compartment model for single infection with pandemic influenza virus. (B) Compartment model for single infection with bacteria. (C) Compartment model for virus – bacterial co-infection in influenza pandemics. See Table 1 and Table 2 for definition of the variables and parameters, and see the text for more details about the model description.

Considering a single homogenous population with no immunity to a novel pandemic strain, we assume that hosts are of four states with respect to the influenza infection, susceptible (X), asymptomatically infected (YF_A_), symptomatically infected (YF_S_) and recovered (ZF) (Figure 1A). The variables, X, YF_A_, YF_S_, ZF and those in the models to follow are both the densities of hosts of each of these states as well as their designations. The population size (N) is the sum of densities of all compartments. These and the other variables of this model and the models to follow as well as their parameters are separately defined in Table 1 and Table 2.

**Table 1:** Variables in the influenza virus – bacterial co-infection model.

Variables	Definition
X	Number of people susceptible to both influenza virus and bacteria
YF_A_	Number of people with asymptomatic influenza infection but not colonized with bacteria
YF_S_	Number of people with symptomatic influenza infections but not colonized with bacteria
ZF	Number of people have recovered from influenza infection
YB	Number of people colonized with bacteria and susceptible to influenza virus
YBF_A_	Number of co-infected people who are colonized with bacteria first then acquire asymptomatic influenza infection
YBF_S_	Number of co-infected people who are colonized with bacteria first then acquire symptomatic influenza infection
YF_A_B	Number of co-infected people who are asymptomatically infected with influenza first and then acquire bacterial colonization
YF_S_B	Number of co-infected people who are symptomatically infected with influenza first and then acquire bacterial colonization
YP	Number of people who develop secondary bacterial pneumonia
ZFYB	Number of people who have recovered from influenza infection but are still colonized with bacteria.
N	Total number of population

**Table 2:** Parameters in the influenza – bacteria co-infection model.

Symbol	Meaning	Base case	Assumptions/References
R_E_	Effective reproductive number for pandemic influenza virus	1.8	Based on Refs. [37], [38], [39] Can be reduced with antiviral interventions
s_F_	Proportion of newly influenza-infected hosts who have typical influenza symptoms	40%	Although 66.9% of influenza infection results in some symptoms [22], we decided to use 40% to get an influenza attack rate similar to those observed in 1918 [2]. Additionally, this number is close to fraction of infected people with typical influenza symptoms (like fever) [22] who are more likely to be prophylaxed.
ν_FS_, ν_FB_	Recovery rate per host per day for YF_A_ and YF_B_ hosts	1/4.8	Based on Ref. [22]. Assume ν_FS_ = ν_FB_
β_FA_β_FS_	Transmission rate constant for hosts with asymptomatic and symptomatic influenza infection.	4.967.92	Calculated from R_E_, ν_FS,_, and s_F_. Assume asymptomatic hosts are half infectious as symptomatic hosts (β_FA_ = 0.5*β_FS_).
d_F_	Death rate per host per day directly due to influenza virus among hosts with symptomatic influenza infection	0.00026	**Virulence parameter estimated by calibration**
p_B_	Prevalence of bacterial colonization before the pandemic	40%	The prevalence of pneumococcal colonization was 40% in 1918 [26], [27], [28]. Varied for different scenarios today
β_B_,	Transmission rate constant for bacteria	ν_B_/(1−p_B_)	Assume bacterial transmission before the pandemic is at equilibrium, thus β_B_ = ν_B_/(1−p_B_). Varied based on p_B_.
ν_B_	Recovery rate per host per day for bacterial colonization	1/37	Based on Ref. [23].
δ_FA_	The increase of bacterial acquisition for hosts with asymptomatic influenza infection	1	Assume asymptomatic influenza infection does not increase the susceptibility to bacterial colonization
δ_FS_	The increase of bacterial acquisition for hosts with symptomatic influenza infection	4	Based on an animal study showing that influenza infection increased the susceptibility of ferrets to pneumococcal acquisition [46].
σ_FA_	The increase of transmission of bacteria for hosts with asymptomatic influenza infection	1	Assume asymptomatic influenza infection does not increase bacterial transmission
σ_FS_	The increase of transmission of bacteria for hosts with symptomatic influenza infection	3.5	Based on a human study testing the dispersal *Staphylococcus aureus* after experimentally infected with rhinovirus [20].
ν_BFS_, ν_BFA_, ν_FSB_, ν_FAB_	Recovery rate per host per day for YBF_S_, YBF_A_, YF_S_B and YF_A_B, respectively	4.8d	Assume equal to ν_FS_ and ν_FB_ because the duration of influenza infection is much shorter than the duration of bacterial colonization.
α_BFA_, α_FAB_	Risk of secondary bacterial for YBF_A_ and YF_A_B	0	Assume people with asymptomatic influenza infections do not develop secondary bacterial pneumonia.
α_BFS_α_FSB_	Risk of secondary bacterial for YBF_S_ and YF_S_B.	**3.6%** **14.4%**	**Virulence parameters estimated by calibration**. Assume α_FBS_ = 4 α_BFS_ in the base case but also consider two extreme conditions: (i)α_FSB_ = α_BFS_; (ii)α_FSB_>α_BFS_ = 0. These numbers are reduced by 45% in countries with PCV program for children.
ν_P_	Recovery rate per host per day for secondary bacterial pneumonia	10d	Based on Ref. [43].
c_P_	Case fatality rate of secondary bacterial pneumonia	30%	Based on Ref. [2].
f_T_	Fraction of symptomatic flu patients treated with antibiotics	0–100%	Varied for different scenarios
c_PT_	Case fatality rate of secondary pneumococcal pneumonia for patients treated with antibiotics	10%	Based on Ref. [49], [61].
f_P_	Fraction of symptomatic flu patients prophylaxed with antibiotics	0–100%	Varied for different scenarios
Ρ	The efficacy of antibiotic prophylaxis in reducing bacterial acquisition	78%	Based on based on a clinical trial testing the effect of short-course, high-dose oral amoxicillin therapy on pneumococcal carriage [40].
Γ	The efficacy of antibiotic prophylaxis in clearing pneumococcal colonization	72%	

Both the YF_A_ and YF_S_ hosts are infectious, with transmission rate constants, β_FA_ and β_FS_ and a fraction, s_F_ (0≤s_F_≤1) of newly infected hosts are symptomatic. Transmission occurs at rates proportional to product of X and λ_F_, where λ_F_ is the sum of the products of the proportions of infected hosts and the corresponding transmission rate constants 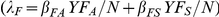. YF_A_ and YF_S_ hosts enter the recovered state (ZF) at rates ν_FA_ and ν_FS_ per host per day. In this, like most compartment models, virulence is reflected in the mortality rate. We assume symptomatically infected hosts (YF_S_) have a death rate directly due to primary influenza infection d_F_ per host per day. The duration of the infections and thereby the amount of time available for transmission are the reciprocals of these rates, for example, symptomatic host, YF_S_, remains infected for 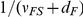 days. The birth rate and influenza-independent death rate are neglected in our model.

#### ii) Single infection with bacteria

Given the variety of pneumococcal serotypes and other bacterial pathogens, we assume that there is no immunity to bacterial colonization. As a result, our model for bacterial transmission only contains two compartments: susceptible (X) and colonized (YB) (Figure 1B). YB hosts are infectious with a transmission rate constant β_B_ and are spontaneously cleared at a rate of ν_B_ per host per day. In this model, we neglect the mortality due to the bacterial infection alone.

#### (iii) Virus – bacterial co-infection

For co-infection we separately consider hosts that are infected by both bacteria and virus and the order at which they are infected, bacteria first or virus first, YBF_A_, YBF_S_, YF_A_B and YF_S_B, respectively (Figure 1C). For example YBF_A_ represents hosts that are first colonized with bacteria and then asymptomatically infected with influenza virus. In this way we can allow for different rates of transmission and rates of recovery of the different jointly infected hosts. The purpose of making this distinction rather than considering only one class of joint infection is to account for the observations made with animal experiments. The likelihood of mortality is different in hosts first infected with the influenza virus than those first infected with the bacteria responsible for the pneumonia [10], [11].

A YBF_A_ or YBF_S_ host can be produced by a YB host encountering one of the influenza infected hosts, YF_A_, YF_S_, YBF_A_, YBF_S_, YF_A_B, and YF_S_B. Similarly, a YF_A_B or YF_S_B host can be produced by a YF_A_ or a YF_S_ host being infected by a host carrying bacteria, YB, YBF_A_, YBF_S_, YF_A_B, YF_S_B, YP or ZFYB. We assume that influenza – infected hosts, YF_A_ and YF_S_, are more likely to acquire bacterial colonization than influenza – free hosts when they encounter bacteria [15], [16], [17]. Therefore, a YF_A_ or YF_S_ host can be infected with bacteria at rate of δ_FA_×λ_B_ or δ_FS_×λ_B_, correspondingly, where δ_FS_ and δ_FA_ are constants ≥1 and λ_B_ is the sum of the products of the proportions of colonized hosts and the corresponding transmission rate constants (see Appendix S1 for the equations). We also assume that co-infected hosts can transmit bacteria more efficiently than influenza – free hosts [18], [19], [20], [21]. For example, co-infected hosts with symptomatic influenza (YBF_S_ and YF_S_B) can transmit bacteria with a transmission rate constant σ_FS_×β_B_ (σ_FS_≥1). Similarly, YBF_A_ and YF_A_B hosts have a transmission rate constant σ_FA_×β_B_ (σ_FA_≥1) for bacteria. On the other hand, we assume that the co-infected hosts have the same transmission rate constant for influenza virus, β_FA_ or β_FS_, as YF_A_ or YF_S_ hosts, depending on whether their influenza infections are symptomatic or not.

The four different co-infected host populations YBF_A_, YBF_S_, YF_A_B and YF_S_B, leave their states at rates ν_BFA_, ν_BFS_, ν_FAB_, and ν_FSB_ per host per day, respectively. Fractions of these co-infected hosts, respectively α_BFA_, α_BFS_, α_FAB_, and α_FSB_ (0≤αs≤1) develop secondary bacterial pneumonia (YP) and the remainder enter state designate ZFYB. In this state individuals have recovered from influenza, but are still colonized with bacteria because we are assuming the duration of infection and infectiousness for the influenza virus is much shorter than for the bacteria [22], [23]. We also assume that jointly infected hosts, YBF_S_ and YF_S_B have an additional death rate from primary influenza infection (d_F_) as do the host symptomatically infected solely with the influenza virus, YF_S_. Hosts with secondary bacterial pneumonia (YP) leave their compartment at rate ν_P_ per host per day. The case fatality of secondary bacterial pneumonia is c_P_ (0≤c_P_≤1 ) and those who survive enter the ZFYB state. Hosts who recover from influenza infection (ZF and ZFYB) are assumed to have long-term immunity to infection with this virus, do not return to the naïve uninfected host state X. On the other hand, we assume that immunity to influenza does not make these recovered ZF hosts any more refractory to bacterial colonization than X hosts.

#### iv) Co-infection model with antibiotic treatment and prophylaxis

**Figure 2:**
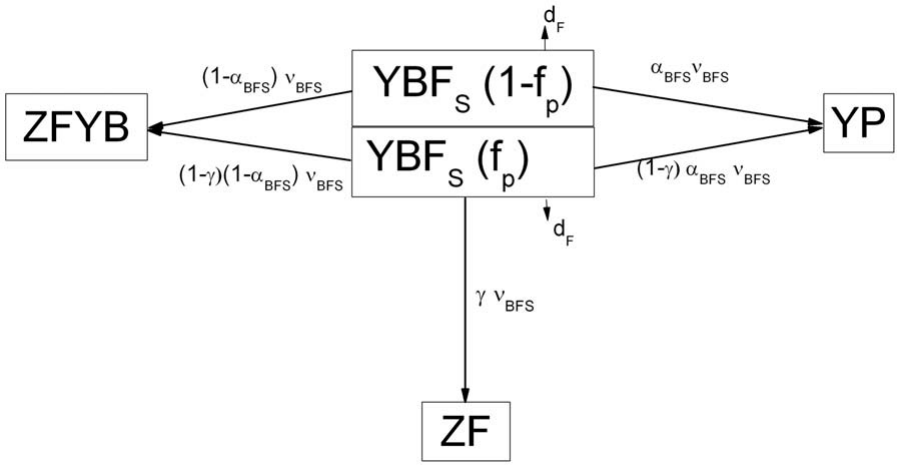
Diagram for how antibiotic prophylaxis is modeled for YBF_S_ hosts. See the text and associated tables for more details.

Antibiotics would be used in two ways. One is to treat patients with secondary bacterial pneumonia. We assume that a fraction (f_T_) of patients with secondary pneumonia, YP, will be treated with antibiotics. The treated people have a lower probability of death (case fatality), c_PT_ and their bacterial colonization is eliminated after treatment. The other way antibiotics would be used is for prophylaxis of hosts with symptomatic influenza to prevent secondary bacterial pneumonia. We assume that prophylaxis is empiric without distinction about whether the prophylaxed host has bacterial colonization or not. Thus, a fraction, f_P_ (0≤f_P_≤1) of YF_S_ and YBF_S_ are prophylaxed with antibiotics. We assume that prophylaxed YF_S_ hosts have a lower probability of acquiring bacterial colonization once they encounter hosts carrying bacteria than unprophylaxed YF_S_ hosts. This efficacy of reducing susceptibility to colonization is represented by ρ (0≤ρ≤1). Therefore, YF_S_ hosts enter YF_S_B at a rate 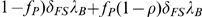. For the prophylaxed YBF_S_ hosts, we assume that the efficacy of prophylaxis to clear the bacterial colonization is γ, and those who clear their bacterial colonization would move to the ZF state. In the remaining (1 - γ), the prophylaxed hosts are still colonized with bacteria and we assume these individuals have the same risk of developing secondary pneumonia as unprophylaxed YBF_S_ hosts. Therefore, (1 - γ) of the prophylaxed YBF_S_ hosts may develop secondary bacterial pneumonia with a probability of α_FBS_ or move to the ZFYB state. We assume that the prophylaxed hosts have the same additional death rate from primary influenza infection (d_F_) as YF_S_ hosts. In Figure 2, we illustrate how antibiotic prophylaxis is modeled for YBF_S_ hosts.

As in other compartment models, the change in the density of each host state is represented by a differential equation. In Appendix S1, we present the complete set of differential equations for this co-infection, treatment and prophylaxis model. For the numerical solutions employed to explore its properties we use Berkeley Madonna™ 8.3 copies of this program are available on www.eclf.net.

### Parameterization

Although our model is general and appropriate for most bacteria responsible for respiratory infections, for our numerical analysis of bacterial elements of the properties of this model we use parameters estimated for *Streptococcus pneumoniae* because pneumococci appear to be single most significant bacteria responsible for secondary infections in 1918, and the necessary epidemiological data seem to be most available for the pneumococci. The values or ranges of values of the parameters used in our models, as well as the sources of justification for these estimates are listed in Table 2.

The parameter d_F_ per host per day is the death rate (virulence) of the 1918 virus for symptomatic infected hosts in the absence of bacterial co-infection. The corresponding virulence parameters for co-infected hosts to develop secondary bacterial pneumonia are, α_BFA_, α_FAB_, α_BFS_, and α_FSB_ for the YBF_A_, YF_A_B, YBF_S_, and YF_S_B host, respectively. We assume that asymptomatic influenza infections do not lead to bacterial pneumonia (α_BFA_ = α_FAB_ = 0). For symptomatic influenza infections, we allow for the possibility that influenza infection preceding pneumococcal colonization results in a higher risk bacterial pneumonia than bacterial colonization preceding influenza infection as the base case (α_FSB_ = 4 α_BFS_) [10], [11]. To explore the sensitivity of the dynamics to this assumption, we also consider situations where α_FSB_ = α_BFS_ and where α_FSB_>α_BFS_ = 0. The values of the virulence - specific parameters for the 1918 virus (d_F_, α_BFS_, and α_FSB_,) are calculated by determining the parameter conditions under which the co-infection model best accounts for the excess all-cause mortality in the New York City during the fall and winter wave of the1918 pandemic (5.3 per 1000) [24], [25]. For this we assume that 7% of this excess mortality was caused directly by virus, with the remaining 93% due to bacterial pneumonia [7] and that pneumococcus was responsible for 71% of the bacterial pneumonias [9].

Given the major role played by the pneumococcus in pneumonia mortality during the 1918 pandemic, the likelihood of an infection with a virulent pneumococcus immediately after influenza becomes a critical risk for pneumonia. In 1918, it would seem that the likelihood of acquiring a new pneumococcus whilst suffering from influenza was greater than it is at present. The prevalence of pneumococcal carriage in adults in 1918 was ∼40% [26], [27], [28], whilst in contemporary populations in developed countries this carriage rate is less than 10% or even less than 5% [29], [30], [31], [32]. It should be noted, however, that pneumococcal prevalence in adults is still very high in some developing countries, such as The Gambia where a 40% carriage has been reported [33]. Another difference between 1918 and today is the current widespread use of the pneumococcal conjugate vaccine (PCV) in children in developed countries, which has reduced the incidence of invasive pneumococcal disease and non-bacteremic pneumonia in all age group by approximately 45% [12], [34], [35]. In its current form our model does not specifically account for the dynamics of a PCV (or influenza) vaccination program. We can, however consider the consequences of vaccination for PCV in one of two ways, by its affect on the rate of transmission, or by its effect on the incidence of secondary bacterial pneumonia by people manifesting the symptoms of influenza. Because of the dearth of data on the serotypes of *S. pneumoniae* responsible for the pneumonias in the 1918 pandemic, to account for the wide spread use of the PCV we assume vaccine reduces the 1918 estimates of α_FSB_ and α_BFS_ by 45% [12], [34], [35]. The transmission rate constant of pneumococcus is not changed because its value depends on the equilibrium pneumococcal prevalence, which has not changed since the introduction of PCV, presumably because of serotype replacement in the nasopharynx [36] (Table 2).

### An overview of the analysis

After using our model to estimate values of the three virulence parameters of the 1918 influenza virus, we predict the incidence of pneumococcal pneumonia (IPP) under different scenarios about the prevalence of pneumococcal colonization at the start of a pandemic with an 1918-like influenza virus and different assumptions about the order of infection. We then investigate the extent to which antibiotic treatment for patients with secondary pneumonia can reduce the incidence and mortality of pneumococcal pneumonia. Finally, we consider the effect of antibiotic prophylaxis for patients with symptomatic influenza on reducing IPP and the pneumococcal prevalence. In this last analysis we explore the number of symptomatic influenza patients needed to be prophylaxed with antibiotics to prevent one case of pneumococcal pneumonia as the Number Needed to be Prophylaxed (NNP).



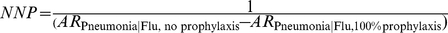
Where AR_Pneumonia|Flu, no prophylaxis_ and AR_Pneumonia|Flu, 100% prophylaxis_ are the attack rates of secondary pneumococcal pneumonia among patients with symptomatic influenza given no prophylaxis and 100% prophylaxis, respectively.

We calculate NNP for different prevalences of pneumococcal colonization in populations with and without PCV programs. We also consider a range of values of the effective reproductive number of influenza (R_E_) [23], because the transmission of influenza virus could be mitigated by other interventions, such as antiviral prophylaxis or influenza vaccines. In our analysis, we are primarily interested in the incidence of pneumococcal pneumonia rather than just the mortality rate. The reason is that the mortality rate reflects factors not considered in the model, like the quality of care or age of the patient. The incidence is also important as it reflects the number of people who need medication and hospitalization. To initiate these simulations, we assume that at the start of the pandemic, a single YFs host is introduced into populations of 1,000,000 people who are wholly susceptible to influenza and different prevalences of pneumococcal carriage. We explore the sensitivity of the predicted NNPs by varying the central parameters by ±10% and generating a tornado plot.

## Results

### Predicting and learning (estimating parameters) from the past

We open our analysis of the properties of this model by exploring its ability to account for observations made in the 1918 pandemic, based on independent estimates of its parameters.

#### The 1918 influenza attack rate

At equilibrium, the fraction of population infected with influenza depends solely on the effective reproductive number R_E_ (roughly the number of secondary infections caused by a single infectious individual entering that population). When R_E_ = 1.8, the estimated value [37], [38], [39], in accord with our model 73% of the population would be infected with the virus. If we assume that 40% of these infected people (s_F_) have typical influenza symptoms (see Table 2), the influenza attack rate would be 29%, which is close to that observed in the 1918 pandemic in the United States [2].

#### The virulence parameters

Assuming the excess mortality rate data for the 1918 pandemic in New York City, the above estimates of the influenza attack rate, and the other parameters in range of those in Table 2, using our co-infection model we determine the best fitting values of the three virulence parameters. We estimate the death rate due to the influenza virus alone, d_F_, to be 0.00026 per day. The magnitudes of probabilities of developing secondary pneumonia by coinfected people, α_FSB_ and α_BFS_, depend on the order of the infections. If we assume a prior symptomatic influenza infection increases the probability of pneumococcal pneumonia (α_FSB_ = 4 α_BFS_), α_FSB_ and α_BFS_ are respectively 14.4% and 3.6%. If the order of co-infection does not matter (α_FSB_ = α_BFS_), the risk of secondary pneumonia for the co-infected hosts is 6.7%. In another extreme case, co-infected hosts who are first colonized with bacteria do not develop secondary pneumonia (α_BFS_ = 0), the probability of developing secondary pneumonia for influenza first infection YF_S_B hosts (α_FSB_) is 23.0%.

### Anticipating the Future

#### The effects of pneumococcal carriage prevalence

**Figure 3:**
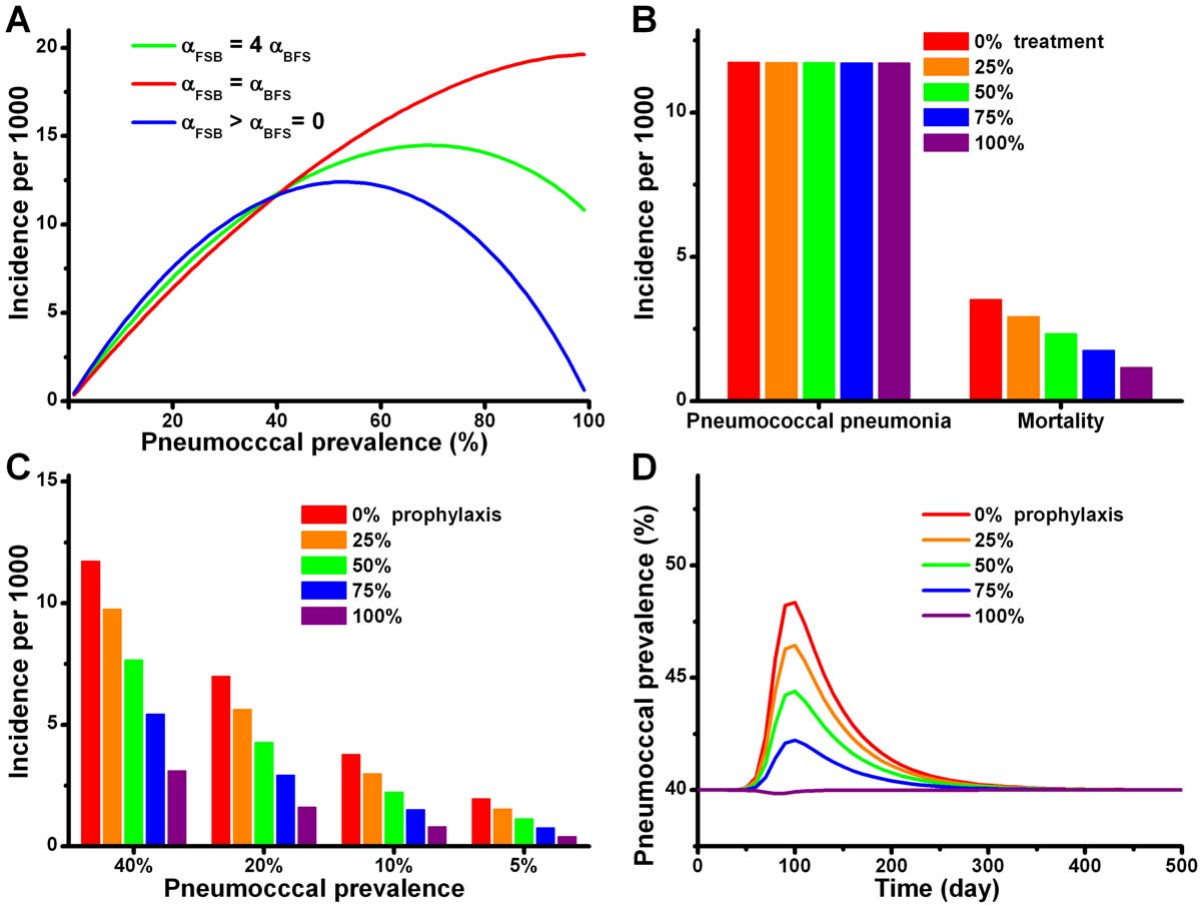
Modeling results. (A) The predicted incidence of pneumococcal pneumonia in a 1918-like influenza pandemic under different initial prevalence of pneumococcal colonization and three assumptions regarding the relationship between α_FSB_ and α_BFS_. (B) The predicted mortality and incidence of pneumococcal pneumonia in a 1918-like pandemic when 0%, 25%, 50%, 75% and 100% of pneumonia patients were treated with antibiotics and the initial pneumococcal carriage was 40%. (C) The predicted incidence of pneumococcal pneumonia in a 1918-like pandemic when 0%, 25%, 50%, 75% and 100% of patients with symptomatic influenza infection received antibiotic prophylaxis under different initial pneumococcal prevalence. (D) The predicted prevalence of pneumococcal colonization during the progress of a 1918-like influenza pandemic when 0%, 25%, 50%, 75% and 100% of patients with symptomatic influenza infection received antibiotic prophylaxis.

Using baseline values of the parameters shown in Table 2, we estimate the incidence of pneumococcal pneumonia (IPP) for a future pandemic due to a 1918-like virus under different assumptions about the prevalence of pneumococcal colonization and the virulence of different orders of co-infection. The results of our analysis are presented in Figure 3A. If there is no order effect, α_FSB_ = α_BFS_, IPP increases monotonically with the pneumococcal prevalence. If there is an order effect, the IPP increases when the prevalence of pneumococcal carriage is low but declines when the prevalence of carriage is high. The reason for this is that fewer people acquire new pneumococcal colonization during the pandemic. However, with respect to the IPP, these three assumptions yield very similar estimates when the prevalence of carriage is within the realistic range (≤40%). Based on this prediction, we restrict the following analysis to a single situation (α_FSB_ = 4 α_BFS_). When the initial prevalences of carriage are 5%, 10%, 20% and 40% the predicted IPPs are, respectively 1.96, 3.78, 7.00 and 11.74 per 1000 population. The mortality caused by primary viral infection does not vary with different pneumococcal prevalence and is approximately 0.37 per 1000 population.

#### Antibiotic treatment

We assume that antibiotic treatment reduces the case mortality rate of pneumococcal pneumonia from 30% to 10% (see Table 2). In Figure 3B we plot the anticipated incidence and mortality due to pneumococcal pneumonia for a 1918-like influenza pandemic as a function of the fraction of the treated patients with secondary pneumonia assuming 40% carriage. These results suggest that although widespread antibiotic treatment for pneumonia would significantly reduce mortality, it would have little effect on the IPP. The reason for this is that people with active pneumonia represent a small fraction of the individuals colonized with these bacteria and thereby responsible for their transmission. Thus, although treatment eliminates colonization as well as increases survival, its effect at the population level is anticipated to be small.

#### Antibiotic prophylaxis

In Figure 3C we consider the anticipated effects of antibiotic prophylaxis on the IPP for different fractions of symptomatic influenza patients receiving these drugs prior to the onset of pneumonia. We make this calculation for different initial prevalences of pneumococcal carriage. In this analysis we are assuming that the efficacy of antibiotic prophylaxis for reducing the susceptibility to bacterial colonization and clearance given colonization are respectively 78% and 72% [40]. As would be anticipated intuitively, antibiotic prophylaxis can substantially reduce the IPP. For example, with these parameters, 40% carriage and 75% of people with symptomatic influenza prophylaxed, the IPP would be reduced by more than 50%, relative to that anticipated in the absence of prophylaxis.

To illustrate the consequences of this intervention, we consider the predicted IPP and the NNP (number needed to be prophylaxed) to prevent one case of pneumococcal pneumonia. We consider this for countries with and without PCV programs and for different effective reproductive number (R_E_), see Table 3 and Table 4. When the R_E_ is 1.8, the estimated NNP to prevent one case of pneumococcal pneumonia in countries without PCV program are 188.6, 98.8, 54.4 and 33.9 when the initial prevalences of pneumococcal carriage are respectively, 5%, 10%, 20% and 40%. The IPP is anticipated to be reduced by approximately 45% and the NNP increased by approximately 81% in countries with a PCV program relative to those without. The R_E_ has marked effect on the estimated IPP, but the NNP is only slighted affected by the R_E_. In coutries with a pneumococcal prevalence of 40%, no PCV program and no antiviral interventions to reduce R_E_, the pandemic would not be very different from that of 1918 pandemic: the estimated IIP is 11.74 per 1000 and the NNP 33.9. On the other hand, in countries with only 5% pneumococcal prevalence and a PCV program, the estimated IPP is 1.08 per 1000 and NNP is 343 when the R_E_ is 1.8. If R_E_ is reduced to 1.2, e.g. by antiviral prophylaxis or influenza vaccines, the esimtated IPP would be reduced to 0.40 per 1000 and the NNP 403.6.

**Table 3:** The estimated incidence of pneumococcal pneumonia (IPP) per 1000 in countries with and without a PCV program under different pneumococcal prevalence and effective reproductive number (R_E_).

	R_E_ = 1.8		R_E_ = 1.5		R_E_ = 1.2	
Pneumococcal carriage	No PCV	PCV	No PCV	PCV	No PCV	PCV
5%	1.96	1.08	1.47	0.81	0.73	0.40
10%	3.78	2.08	2.85	1.57	1.42	0.78
20%	7.00	3.85	5.31	2.92	2.68	1.47
40%	11.74	6.45	9.05	4.98	4.68	2.57

**Table 4:** The estimated number needed to be prophylaxed to prevent one case of pneumococcal pneumonia (NNP) in countries with and without a PCV program under different pneumococcal prevalence and effective reproductive number (R_E_).

	R_E_ = 1.8		R_E_ = 1.5		R_E_ = 1.2	
Pneumococcal carriage	No PCV	PCV	No PCV	PCV	No PCV	PCV
5%	188.6	343.0	201.6	366.5	222.0	403.6
10%	98.8	179.6	105.1	191.1	115.1	209.3
20%	54.4	99.0	57.4	104.4	62.1	112.9
40%	33.9	61.7	35.2	63.9	36.9	67.1

In Figure 3D, we follow the temporal changes in the prevalence of pneumococcal colonization during the course of the pandemic with different fractions of the population prophylaxed and an initial pneumococcal carriage prevalence of 40%. In the absence of antibiotic prophylaxis, pneumococcal prevalence gradually increases to 48.5% during the pandemic and then returns to the equilibrium level after the pandemic. Antibiotic prophylaxis would reduce bacterial transmission and thereby the level of pneumococcal carriage during the pandemic.

### Sensitivity analysis

**Figure 4:**
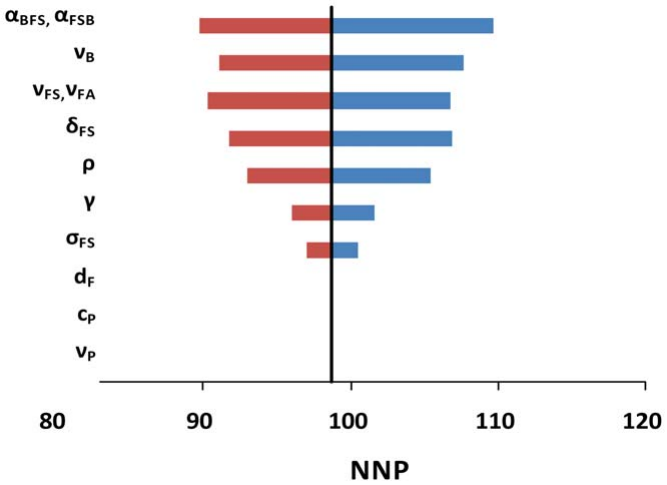
Sensitivity analysis. Tornado plot of number needed to be prophylaxed (NNP) to prevent one case of pneumococcal pneumonia with ±10% changes in parameters when the initial pneumococcal prevalence is 10%.

To deal with the uncertainty of parameter values, we use a tornado plot to explore the sensitivity of our predicted NNP by varying the dominant parameters by ±10% for a situation where the prevalence of bacterial colonization is 10% (Figure 4). The estimated NNP is most sensitive to the risks of secondary pneumonia among the co-infected people (α_FSB_ and α_BFS_). Other influential parameters included the recovery rate for pneumococcal colonization (ν_B_), the recovery rate for influenza (ν_FS_ and ν_FA_), the effect of influenza infection on bacterial colonization and transmission (δ_FS_ and σ_FS_), the efficacies of antibiotic prophylaxis on bacterial transmission and colonization (ρ and γ).

## Discussion

“It's tough to make predictions, especially about the future.”

(Attributed to Yogi Berra but also Neils Bohr)

Were the world confronted with a pandemic due to an influenza virus with a transmission rate, virulence and virulence mechanism similar to that of the 1918 H1N1 virus, would we better off now than we were then? We interpret the results of this theoretical study as support for a positive answer to this question.

Central to our model and this interpretation is the evidence that most of the morbidity and mortality of the 1918 pandemic can be attributed to secondary bacterial infections, primarily pneumonia due *S. pneumoniae* (the pneumococcus). The evidence and arguments in support of this assertion have been presented elsewhere and won't be reviewed here [7], [8], [9], [41], [42], [43], [44], [45]. Also central to our model and interpretation is the premise that respiratory viral infections increase the likelihood of colonization by pneumococci [15], [16], [17], [46] and the rate of transmission of bacteria [18], [19], [20], [21], [46]. Finally we assume that in the course of an influenza pandemic with the transmissibility and virulence of that of 1918, virtually all cases of pneumococcal pneumonia occur in co-infected people.

There are two primary reasons for anticipating substantially lower rates of the bacterial pneumonia responsible for most of the morbidity and mortality of the 1918 influenza pandemic, especially in current developed countries. First, in developed and many underdeveloped countries the prevalence of pneumococcal carriage in adults is substantially lower than it was in 1918 [26], [27], [28], [29], [30], [31], [32]. As a result there would be both lower rates of pneumonia and the infectious transmission of these bacteria. Second is the widespread use of pneumococcal conjugate vaccines (PCV). This vaccine appears to contribute little to the decline in overall prevalence of carriage of these bacteria, due to the replacement of the vaccine serotypes by others [36]. On the other hand, there is good evidence that PCV reduces the likelihood of pneumococcal pneumonia in not only vaccinated individuals but also in the population at large, which is the way we incorporated its widespread use in our analysis. Although some of this population-wide reduction in pneumococcal pneumonia is due to herd immunity [34], [35], this transmission component of a vaccination program is not formally considered in our model. It is, however, implicit in our assumption that the vaccine reduces the incidence of pneumococcal pneumonia by 45%.

In many cases, interventions for infectious diseases that are good for individuals may have little positive and sometimes even may even negative consequences for the collective. The results of our analysis suggest this is going to be the case for antibiotic prophylaxis during a 1918-like influenza pandemic. Because of the relatively small risk of secondary bacterial infections in populations with low and modest prevalence of pneumococcus carriage, antibiotic prophylaxis for all symptomatic influenza patients would have little effect in reducing the incidence of pneumonia in the collective. In accord with our analysis, hundreds of patients with symptomatic influenza would need to be prophylaxed, NNP, to prevent a single case of secondary pneumococcal pneumonia, even in this model which assumes that asymptomatic influenza infection does not increase the susceptibility to bacterial colonization or transmission. If asymptomatic infection can in fact increase bacterial colonization or transmission, antibiotic prophylaxis will be even less effective than predicted by our model because antibiotic prophylaxis in this model targets only symptomatic patients. Our model further does not consider the potentially deleterious impact that mass antibiotic prophylaxis may have on antibiotic resistance. When considering this NNP and contribution of antibiotic use to the ascent of resistance, at the level of the collective, antibiotic prophylaxis for all symptomatic influenza infections would be difficult to justify. This is particularly so when antibiotic treatment for the bacterial pneumonias that do arise in this small minority is a viable alternative to prophylaxis for many.

In this regard, a very different conclusion may be in order for underdeveloped countries where the prevalence of pneumococcal carriage is substantial [33]. Because of the latter, the estimated NNP to prevent a single case of secondary pneumonia would be on the order of 30–35. Unfortunately, associated with high frequencies of pneumococcal carriage in these countries is a dearth of the money needed for the wide spread purchase of prophylactic antibiotics. No matter where, the cost effectiveness of antibiotic prophylaxis would greatly augmented if there were procedures to identify people who are at particular risk of these secondary infections or members of clear risk groups, like people with other co-morbidities. During the 2009 H1N1 pandemic patients aged 6–65 years who carried the pneumococcus in the nasopharynx were at much higher risk of severe pneumonia or death compared to patients without pneumococcal carriage (adjusted odd ratio 126) [47].

Antibiotic treatment of secondary bacterial infections would also be more advantageous to individuals than populations. In accord with our analysis, the treatment of patients with pneumococcal pneumonia would have a negligible affect on the transmission and thereby the frequency of carriage and infection by these bacteria. Unlike prophylaxis, however, the individual benefit of the use of antibiotics for treatment can be considerable and will almost certainly outweigh the cost associated with the promotion of resistance. Indeed, if we consider the mortality of bacteriemic pneumococcal pneumonia before and after the introduction of penicillin, 80% down to 10–15%, [48], [49], which is where it is now [50], antibiotic treatment is of considerable advantage to individuals with this disease.

If, as suggested by the animal model experiments [10], [11], the likelihood of pneumonia in humans is greater when the bacteria follow the virus infection than the reverse, the order of the infection would play an important role in the course of the disease for individuals. Our results suggest, however that this order effect may contribute little to the incidence of bacterial pneumonia for the population at large. As long as the prevalence of carriage is modest, less than 40%, the incidence of pneumococcal pneumonia, IPP, is relatively independent of the order of infection (see Figure 3A). On the other hand, when the prevalence of pneumococcal carriage is greater than 40%, the order of infection becomes increasingly important at the population as well as the individual level. In fact, as the prevalence increases bacterial colonization can be protective if the likelihood of pneumonia is greater when the viral infection precedes the bacterial. That is, as the prevalence of carriage increases, a greater fraction of people infected with the influenza virus would already be colonized with pneumococcus.

As complex as our model might seem, it captures only some of the real complexity of the epidemiology of influenza, bacterial pneumonia and the prevention and treatment of these diseases in human populations. Contrary to what we assumed in our model: (i) Human populations are not homogeneous and have multiple subpopulations. The rates of transmission, prevalence of pneumococcal carriage and the parameters governing course of the infection and co-infection are not going to be the same for all subpopulations. Age, life-style, social contact pattern, local density and physical condition will all contribute to the values of these parameters. Also contributing to this variation is immune state of these hosts due to prior encounters with influenza viruses and pneumococci that are antigenically the same or cross reacting with those encountered during the pandemic. (ii) Pneumococci are not homogenous. There is great deal of genetic variation in *S. pneumoniae* including variation in the capsule structure, their serotype, of which there are 93 at last count [51]. This underlying variation will certainly contribute to individual differences in the infection and carriage parameters as will the extent of coverage by polyvalent, but much less than 93- valent vaccines.

While we can incorporate these other complexities into our model, at this stage we don't see much justification in doing so. There are two reasons for this, one practical and one philosophical. Estimates of the parameters of this extended model are not available. Although we could generate numerical solutions to the large numbers of equations in a more complex and realistic model, without the constraints of parameter values in a realistic range it would be difficult to interpret the implications of the results of this analysis. This interpretation problem would be further confounded by the vast numbers interactions between different elements of this model.

The philosophical justification for not expanding the complexity of these models is their role in this endeavor. In an essay about model building in population biology written more than a half century ago [52], Richard Levins argued that there are three properties of a mathematical model we want to maximize, reality, generality and precision. He postulated that we are only able to maximize two at a time. To address this general question about the morbidity and mortality of a pandemic with a 1918-like influenza virus in contemporary populations, reality and generality are more important than precision. Moreover, because of the relative dearth of estimates of parameters and the problems of interpreting complex models, reality and generality are the best we can achieve at this time.

While our model is general for any combination of directly transmitted viruses and bacteria, we restricted our numerical analysis of its properties to only a single species of bacteria, *S. pneumoniae*. These are not the sole bacteria known to be responsible for bacterial pneumonia during the 1918 influenza pandemic or anticipated to be so in future pandemics. Part of our justification for focusing on pneumococcus in this is by default. Estimates of the necessary parameters are more available for the pneumococcus than other bacteria responsible for pneumonia. Another justification is the relative prevalence of the different species of bacteria responsible for these pneumonias. A review of antemortem cultures from normally sterile sites of pneumonia patients in the 1918 pandemic showed that respectively *S. pneumoniae*, hymolytic Streptococci (Group A Streptococcus) and all other bacteria comprised 71%, 28% and 1% of positive cultures [9].

In contemporary populations the pneumococcus remains the predominant bacterium responsible for community-acquired bacterial pneumonia [53]; group A Streptococci are rare as a source of these pneumonias (0–1%), athough they were commonly associated with measles and influenza outbreaks in the pre-antibiotic era [54], [55], [56]. Postmortem culture studies suggest that *Staphylococcus aureus* pneumonia became a significant source of mortality following influenza in subsequent influenza pandemics and in contemporary seasonal influenza [57], [58], [59]. We suggest that to some extent this observation is the product of sampling bias in the era of antibiotic use. Because *S. aureus* pneumonias are more likely to be fatal than those due to pneumococci and because of concern about the incidence of antibiotic resistance in Staphylococci, these bacteria may be more likely to be cultured in postmortems of antibiotic-treated patients. Most importantly, *S. aureus* pneumonias are primarily nosocomial and less likely to be responsible than pneumococci for the community-acquired pneumonias that are the focus of our model. Be all this as it may, as noted, our model is a general analogue of the epidemiology of viral – bacterial co-infection. By changing the parameter values, it can be applied to any combination of directly transmitted viruses and bacteria.

In this report, we have presented the 1918 influenza as a worst case. In theory, an influenza pandemic could be even more devistating than that of 1918, espectically if antibiotic and vaccine treatable and preventable secondary bacterial infections are not be the major source of mortality. The H5N1 Avian influenza virus has a much higher case mortality rate than the 1918 H1N1 and it is not clear how much of this mortality can be attributed to secondary infections with bacteria [7], [60].

In conclusion, as a consequence of relatively lower prevalence of pneumococcal carriage and intervention with vaccines and antibiotics, the mortality of a pandemic with an 1918- like influenza would be profoundly less in contemporary populations than witnessed in 1918.

## Supporting Information

Appendix S1 **Differential equations for the complete model with co-infection, antibiotic treatment and prophylaxis.**
(DOCX)
