# Supplementary material for: The Anticipated Severity of a “1918-Like” Influenza Pandemic in Contemporary Populations: The Contribution of Antibacterial Interventions
Source: PLoS One. 2012 Jan 23;7(1):e29219. doi: 10.1371/journal.pone.0029219 (PMC3264555; doi:10.1371/journal.pone.0029219)
Supplement: Appendix S1 — Differential equations for the complete model with co-infection, antibiotic treatment and prophylaxis. (DOCX) [file pone.0029219.s001.docx]

**Appendix S1.**

**Differential equations for the complete model with co-infection, antibiotic treatment and prophylaxis.**
